# The use of Macitentan in Fontan circulation: a case report

**DOI:** 10.1186/s12872-017-0567-5

**Published:** 2017-05-22

**Authors:** Polyvios Demetriades, Amir Aziz, Robin Condliffe, Sarah E. Bowater, Paul F. Clift

**Affiliations:** 1 Department of Adult Congenital Heart Disease, Queen Elizabeth Hospital, Birmingham, UK; 2 Pulmonary Vascular Disease Unit, Royal Hallamshire Hospital, Sheffield, UK

**Keywords:** Endothelin-receptor antagonists, Macitentan, Congenital heart disease, Fontan circulation, Pulmonary hypertension, Case report

## Abstract

**Background:**

The Fontan circulation, a result of a palliative procedure in patients with single systemic ventricles, is defined by chronically elevated pulmonary vascular resistance. When traditional heart failure therapies fail, pharmacological agents that reduce pulmonary artery pressures may be used. These include endothelial-receptor antagonists, prostanoids and phosphodiesterase type 5 inhibitors. We report the first use of macitentan, an endothelin-receptor antagonist, in a patient with a Fontan circulation.

**Case presentation:**

We describe the case of a 50 year old female with tricuspid atresia and transposition of the great arteries. Following complex surgery as a child, she subsequently underwent a fenestrated modified atrial pulmonary Fontan operation which was later converted to a total cavopulmonary anastomosis Fontan circulation. Due to failure of various medications to relieve her worsening symptoms, she was commenced on macitentan in April 2016. Few months later, she demonstrated a significant symptomatic improvement and associated increase in her incremental shuttle walking test distance.

**Conclusions:**

Macitentan has slower receptor dissociation kinetics compared to other endothelin-receptor antagonists, leading to enhanced pharmacological activity with promising effects in patients with pulmonary arterial hypertension. The patient we report has shown considerable improvement in exercise capacity following introduction of this medication and thus we suggest further randomised trials to establish the role of different endothelin-receptor antagonists in the management of the Fontan circulation.

## Background

The Fontan circulation results from a palliative surgical procedure, which is performed in patients with a functionally single ventricle cardiac anatomy (i.e. univentricular heart). Venous blood is diverted from the vena cava to the pulmonary artery bypassing the single ventricle. The success of this operation has resulted in an increasing population of adults living longer with congenital heart disease [1].

The Fontan circulation is defined by low cardiac output and elevated central venous pressure. The main limitation of the Fontan circulation is the absence of a sub-pulmonary ventricle. Blood flow through the pulmonary vasculature depends on the gradient between central venous pressure and ventricular end-diastolic pressure, as well as a low resistance to flow across the pulmonary vascular bed (pulmonary vascular resistance) [1].

It remains to be determined whether late deterioration is caused by primarily ventricular failure and increasing end diastolic pressure or if it is due to chronically increased pulmonary vascular resistance resulting in raised end-diastolic pressures and therefore increased systemic venous congestion and reduced cardiac output [2]. There is evidence of pulmonary vascular remodelling in failed Fontan patients [3], which is difficult to predict by catheter based assessment of pulmonary vascular resistance [4].

Eventually there is a decrease in exercise capacity, functional status and an increase in heart failure-related hospital admissions with an increase in mortality [1].

Traditional therapies for heart failure directed at improving function/decreasing afterload may not be as relevant in a circulation in which the primary problem is filling as opposed to emptying the ventricle. Treatments aiding ventricular filling in the Fontan circulation may be beneficial.

Modulating pulmonary vascular resistance could improve cardiac output. Reducing pulmonary vascular resistance improves blood flow across the pulmonary capillaries, which results in a reduced central venous pressure and improved cardiac output. Various pharmacological agents have shown to reduce pulmonary artery pressures such as endothelial receptor antagonists [5] prostanoids [6] and phosphodiesterase type 5 inhibitors [7]. This case report involves the first use of macitentan (an endothelin-receptor antagonist) in a patient with a Fontan circulation.

## Case presentation

We describe the case of a 50 year old Caucasian female patient who was born with tricuspid atresia and transposition of the great arteries. At the age of 4, she underwent a modified Glenn procedure followed by a classical left Blalock-Taussig shunt at the age of 16. Since then, she has had two major cardiac operations. In 1990, at the age of 25, she had a fenestrated modified atrial pulmonary Fontan. Following a very difficult pregnancy 18 years later, she underwent conversion to a total cavopulmonary anastomosis Fontan circulation, modified MAZE procedure and implantation of an epicardial defibrillator with biventricular pacemaker system. Cardiac catheterisation at that time showed a ventricular end diastolic pressure of 16 mmHg and a Fontan chamber pressure of 18 mmHg, giving a transpulmonary gradient of 2 mmHg. Her cardiac medical history was further complicated with arrhythmias requiring ablation in 1998 and episodes of non-sustained ventricular tachycardia in 2014.

This lady has been under regular follow-up by our team since 2008. Various medications have been trialled over the last 8 years due to her progressive reduction in exercise capacity and exertional dyspnoea. She had initially taken part in a trial to test the safety of bosentan in 2010 [5] and at the time had shown good response to the drug with improvement in 6-min walk test. Bosentan was discontinued at the end of the study.

Unfortunately, over the course of the next few years, she continued to deteriorate with worsening breathlessness (NYHA III), four-pillow orthopnoea and paroxysmal nocturnal dyspnoea, ventricular function was preserved. She noticed a significant limitation in everyday activities, requiring social care input and a mobility scooter for outdoor travel. Sildenafil was commenced however this was discontinued shortly after due to symptomatic hypotension.

Cardiac CT scan confirmed non-restrictive Fontan pathway, and normal ventricular function.

In early 2016, bosentan was re-commenced with good symptomatic improvement. Unfortunately on this occasion, this was poorly tolerated with significant side effects (peripheral oedema, pruritus) and thus stopped. On the basis of her symptomatic improvement with bosentan we decided to try an alternative endothelin-receptor antagonist, macitentan to alleviate symptoms.

Macitentan (10 mg once daily) was initiated in April 2016. At 5-month review, the patient reported symptomatic improvement and this was associated with an increase in her incremental shuttle walking test distance from 160 m pre-treatment (prior to bosentan administration) to 200 m post-treatment with macitentan. She has not reported any significant side effects. Her echocardiogram remained unchanged with normal systemic ventricular internal dimensions and good radial systolic function (ejection fraction 55–60%).

## Discussion and Conclusions

This is the first patient with Fontan circulation that has been commenced on macitentan. Macitentan belongs to the family of endothelin-receptor antagonists (ERAs) [8]. Macitentan has significantly slower receptor dissociation kinetics than other ERAs contributing towards an enhanced pharmacological activity compared to the other ERAs [5]. In the randomised-control SERAPHIN study, macitentan significantly improved health-related quality of life in Pulmonary Arterial Hypertension (PAH) patients compared to placebo, and both 3 mg & 10 mg doses of macitentan significantly reduced the risk of the composite endpoint of morbidity and mortality [9].

There is limited published evidence to support the use of pulmonary vasodilator therapy in Fontan circulation failure. A recent systematic review identifed improvements in exercise capacity, exercise haemodynamics and ventricular performance following the administration of bosentan and sildenafil in pulmonary hypertension [10]. The TEMPO study was a randomised, placebo-controlled, double-blind study assessing the medium-term effect of bosentan on exercise capacity and functional class together with a safety assessment in Fontan patients. The results were encouraging as there was a significant difference in oxygen consumption, functional class and exercise capacity in the Bosentan group versus the placebo group with no increase in side effect profile [11]. Giardini et al. [7] performed a randomised study assessing the effects of sildenafil on cardiopulmonary exercise testing in Fontan patients in the short term; this study demonstrated that sildenafil improved exercise capacity and haemodynamic response to exercise.

There remain concerns regarding late liver complications in the Fontan circulation, and ERAs have been reported to cause liver dysfunction in a minority of patients. In our own safety study [5] and in the TEMPO study [11] no liver complications were reported and in our patient no liver dysfunction has been recorded. All patients on an ERA are required to have monthly liver function tests performed.

In this case report, both Bosentan and Sildenafil medications were tried, however they had to be discontinued due to intrusive side effects. In view of the promising evidence of the success of macitentan in PAH, this was considered as a potential positive treatment in a patient with Fontan circulation. The patient has shown considerable improvement in exercise capacity following introduction of this medication and thus we suggest further randomised trials to establish the role of ERAs in the management of the Fontan circulation.

## Abbreviations

ERAs: Endothelin-receptor antagonists
PAH: Pulmonary Arterial Hypertension
